# Supplementary material for: Are carbon emissions trading and green financial instruments synergistic? -Comprehensive quantitative research based on content analysis
Source: PLoS One. 2024 Mar 7;19(3):e0298601. doi: 10.1371/journal.pone.0298601 (PMC10919601; doi:10.1371/journal.pone.0298601)
Supplement: S1 Data — (ZIP) [file pone.0298601.s001.zip › Data/地级市Z_F工作报告.zip!/║■▒▒/╧╠─■╩╨/└┤╫╘╣1⁄2╓┌║┼í╛┬φ┐╦╩2╛▌═°í┐_2016.docx]

各位代表：

现在，我代表市人民政府向大会报告工作，请予审议，并请市政协各位委员和其他列席人员提出意见。

一、“十二五”时期经济社会发展及2015年工作回顾

过去的五年是我市经济社会快速发展、城乡面貌发生巨变、人民群众实惠最多的五年。在省委、省政府和市委的坚强领导下，市政府团结和依靠全市人民，主动适应经济发展新常态，大力推进省级战略咸宁实施，解放思想、超前谋划、抢抓机遇、攻坚克难，胜利完成了“十二五”规划确定的主要目标任务，谱写了 “火热蓬勃、亮点纷呈、东风浩荡、香气弥漫”的壮丽篇章。

——经济总量突破千亿。地区生产总值翻一番，达到1035亿元，年均增长11%，增速超过全省平均水平；社会消费品零售总额翻一番，达到408.8亿元，年均增长14.8%；全社会固定资产投资五年累计翻两番，达到4829亿元，年均增长25.6%；一般公共预算收入达到80.12亿元，年均增长28%，是2010年的3.43倍；地方税收达到50.29亿元，年均增长29%，是2010年的2.57倍；全社会劳动生产率由3.55万元/人上升到6.47万元/人，提高近一倍。咸宁由“后发”变“厚发”，跨入中等收入地区行列。

——绿色崛起成效凸显。率先在全国编制绿色崛起规划，空气质量、生态环境指标位居全省前列，森林覆盖率达到49.32%（新标准）。五年来，荣获国家园林城市、国家卫生城市、国家森林城市、全国绿化模范城市、国家旅游标准化示范城市等称号，成为国家生态保护与建设示范区、全国旅游改革创新先行区、全国水生态文明试点市、国家可再生能源建筑应用示范市、国家信用示范市、国家智慧城市试点。向阳湖、富水、青山、大溪成功创建国家湿地公园。

——香城泉都亮点纷呈。连续举办国际温泉文化旅游节，温泉旅游咸宁标准上升为国家标准。十六潭公园、香泉映月、冰雪王国、香城古街、嫦娥广场等城市景观和青少年宫、博物馆、传媒大厦等城市建筑成为咸宁新地标。大型实景神话剧《嫦娥》、民俗风情歌舞剧《时舞?香城恋》开演，提升了咸宁文化品位。“香城泉都”文化成为全省优秀文化品牌。

——区位优势日渐放大。城际铁路在全省最早通车，杭瑞、大广、咸通、咸黄高速建成通车，武汉城市圈外环高速、武深高速咸宁段和嘉鱼长江大桥加快建设，形成了咸宁市区至武汉及周边县市一小时交通圈。主城区外环全线贯通，高速公路县县畅通，长江港口与武汉融通，航空护林站顺利开通，“中三角”枢纽地位初步形成。

——民生事业全面进步。社保、就业、教育等重点民生投入达到632.13亿元，占公共财政支出比重达70%。城镇和农村居民人均可支配收入分别达到23534元、11980元，年均增长11.4%、13.3%，快于地区生产总值增速。坚持每年办好“十件实事”，人民群众幸福感获得感大幅提升。

五年来，我们主要做了以下工作：

（一）积极应对各种挑战，促进经济持续健康发展。面对复杂多变的经济形势和连续多年的旱涝灾害，我们保持定力、应急谋远，及时出台措施，努力化解风险。坚持定期拉练检查，推进重大项目建设，保持了经济快速增长的势头。建立了6604个、总额3.25万亿的项目储备库，102个项目列入省重大专项计划，47个项目进入农发行政策性融资项目库。设立10亿元重大产业发展基金，实施“小钢炮”计划，设立“无微不至”微笑基金，推进金融支持县域经济发展“五个一工程”。成立城发集团，进一步理顺咸宁高新区体制，打造市级经济主平台、主阵地。出台支持绿色产业发展九条措施，推动智能机电等高新产业发展。加快培育“互联网+”消费热点，扩大旅游、养老、文化创意等服务性消费。出台支持城区房地产发展政策，促进住房消费健康发展。五年来，市城区商品房总成交5.74万套、641万平方米、223.3亿元。

（二）加快转型升级步伐，促进产业结构逐步趋优。以“一工程三计划”为抓手，实施工业崛起战略。规上工业企业879家，增加值520亿元，年均增长20.3%。六大支柱产业占规上工业总量74.7%，年均增长22.8%。九大产业纳入全省重点成长型产业集群。高新技术企业99家，增加值80亿元，年均增长39.6%，占GDP比重8%。中航科工、中电华基、中天引控、奥瑞金制罐等项目成功落户，华润电厂二期、金盛兰冶金、南玻玻璃、兴民钢圈、祥天空气能、亚细亚陶瓷等重点项目建成投产。咸宁核电前期工作进展顺利。以工业化理念谋划农业，楠竹、茶叶、油茶三大“百亿产业”发展壮大。粮食总产量过百万吨，实现“十二连增”。农业总产值296亿元，农产品加工业产值与农业总产值之比2.4:1，高于全省平均水平。建成农业产业化板块基地366万亩，省级龙头企业53家，“三品一标”425个，咸宁砖茶销量占全国30%以上。实施旅游跨越战略。累计旅游总收入700亿元，年均增长28.7%。共接待国内外游客1.36亿人次，是“十一五”期间的4.3倍。旅游经济指标位居全省第五，跨入第一方阵。电子商务发展迅猛，通山、赤壁纳入全国电子商务进农村综合示范试点，绿购网成为全省电子商务示范创建企业。商贸物流加快发展，沃尔玛、新世界百货、大润发等知名企业入驻。三次产业比重调整为17.5:49:33.5。

（三）加大改革开放力度，促进创新驱动持续发力。推出“1+N”改革行动，持续释放改革红利。围绕“三张清单”，推动政务服务“二次创新”。市级行政审批和服务事项精简55.2%，涉企收费项目由202项减少到92项。全面推行“三证合一”、“一照一码”企业登记制度，市场主体达到20.3万户。政府机构改革稳步实施，国有企业改制、事业单位分类改革有序推进。财政预算体系建设全省领先，零基预算、“营改增”、政府性债务管理等有效推进。通城、崇阳公立医院改革先行先试，嘉鱼被确定为全国减肥增效试点县。市域金融工程有力推进，成立金投集团，设立红土成长创投基金，启动金融信息港建设。通山、崇阳被确定为省级县域金融工程示范试点县。4家企业挂牌“新三板”，43家企业进入区域性股权交易市场，100家企业挂牌展示板，“通山板块”率先成为省“四板”县域板块。在全国首创“政银保集合贷”，连续获评全省金融信用市。大力推动科技创新，21项科技成果获省部级以上奖励，湖北省首个科技成果转化示范基地成功落户。2015年专利授权总量增幅和企业专利授权量增幅居全省市州第一。中国驰名商标总数达17件。积极推进香城创业计划。设立2.1亿元创业扶持基金，建设25个创业孵化基地，孵化创业实体3600余个，获批省级众创空间3家。实施人才保障战略。精心组织“招硕引博”，博士后工作站、院士专家工作站分别达到12家、31家。深化开放合作。20家世界500强企业落户投资，与100多个国家和地区建立直接贸易往来，缔结国际友好城市2个。累计引进项目2820个，到位资金2728.7亿元。累计实际利用外资7.5亿美元、出口总额16亿美元。推进区域协作，与岳阳、九江、安庆达成《咸宁共识》。成功举办2015国际茶业大会。

（四）加快推进城乡统筹，促进区域发展更加协调。率先在全省编制全域新型城镇化总体规划，构建“12255”空间格局。大力实施城市提质工程。建成绿地面积超过2200公顷，城市绿化覆盖率达40%。城市精细化管理进一步加强，数字化“大城管”格局初步形成。推动城市空间拓展，“四城四区”建设加快。咸安旧城改造、嘉鱼文体会展中心、赤壁生态新城、通城银山广场、崇阳“一河两岸”景观工程、通山通羊河景观改造加快建设。全市城市建成区面积突破154平方公里，全市城区人口突破120万，城镇化率49.6%。县域经济提速增效，“北三县”沿江产业带和“南三县”绿色经济带竞相发展。县域公共财政收入年均增长28%，嘉鱼、咸安地方公共财政总收入突破10亿元，赤壁突破20亿元。统筹城乡一体化发展，嘉鱼潘家湾镇列入全省“四化同步”试点镇，8个村列入全国首批“传统村落保护名录”，60个村被评为省级新农村建设示范村。争取水利投资37.9亿元，完成373座病险水库除险加固和17个中小河流治理，解决53.37万农村居民和14.44万师生饮水安全。咸安、嘉鱼、崇阳获批省级现代农业示范区。“三万”活动全面开展，共兴修当家塘1.2万口，修建垃圾池2万多个，加宽农村通村公路1825公里。幕阜山扶贫脱贫扎实推进，累计整合资金56.2亿元，实现19.1万人脱贫。

（五）坚持绿色发展引领，促进生态优势更加彰显。实施生态持续战略，出台《加强生态文明建设的意见》等26项政策措施，推动绿色崛起行动计划。坚持“显山露水见园林”绿色城市建设思路，生态城市竞争力排名全国第八。大力开展“绿满鄂南”行动，五年植树造林176.2万亩。成立咸宁市排污权储备中心、低碳产业文化服务中心，全省首个碳排放交易试点落户通山。先后实施生态环境建设“十大工程”、“六大计划”，省定节能减排任务圆满完成。加大重点流域水生态综合整治力度，国控、省控地表水监测点和跨市、县级以上城镇集中式饮用水源地水质达标率100%，功能区水质达标率96.9%。重拳整治非煤矿山，“三禁三治”活动取得成效。实行环境保护“一票否决制”，强化“负面清单”管理和区域环境治理，严格执行项目引进“五个一律”制度。建立农村生活垃圾“336处理”、污水处理1+N工程模式和农村环保站等监督管理长效机制，实施83个村庄农村环境连片整治。成功创建2个国家级生态村、10个省级生态乡镇、67个省级生态村。积极构建全民参与的绿色行动体系，生态文明教育普及率90%以上。

（六）大力实施民生工程，促进发展成果全民共享。五年新增城镇就业23.4万人，城镇登记失业率控制在2.7%以内。五项社会保险参保154.9万人次，综合参保率达98%。开工建设保障性住房8.94万套，改造农村危房3.5万户。提高城乡低保标准，惠及19.79万人。发放救灾资金2.35亿元，救助群众150多万人次。加快义务教育均衡发展，6个县市区全部通过省级以上评估验收，市实验外国语学校建成开学。建成219所农村标准化寄宿学校，462所中小学办学条件整体改善，九年义务教育巩固率达99%，高中教育毛入学率达90.3%。基层医疗卫生服务体系逐步完善，新型农村合作医疗、村级卫生室标准化、药品零差率销售实现全覆盖。“感动咸宁”、“最美系列”人物评选凝聚强大社会正能量，“一五一十”工程叫响全国。深入推进“平安咸宁”建设，社会治安防控体系进一步健全，城区电子监控实现全覆盖，易肇事肇祸精神病人集中救治和吸毒人员强制收戒经验全国推广，网格化服务管理运转良好，荣获“中国城市管理进步奖”。“六五”普法顺利完成，依法治市稳步推进。进一步加强信访、食品药品安全监管、质量监督、安全生产工作，全市未发生较大以上群体性事件和安全生产事故，社会大局和谐稳定。

国防动员和后备力量建设进一步加强。对口支援新疆、建始县工作深入推进。妇女儿童、青少年、关心下一代、老龄、残疾人等事业全面发展。对台、侨务、外事、科普、保密、档案、方志、统计、气象、人防、民族宗教、防震减灾、决策咨询等工作取得新的成绩。

过去五年，我们在市委的领导下履职尽责，自觉接受人大法律监督、工作监督和政协民主监督。办理人大代表建议304件、政协委员提案634件，办结率100%，满意率95%以上。认真贯彻中央八项规定、省委六条意见和市委七项要求，着力整改“四风”突出问题。深入开展党的群众路线教育实践活动，自觉践行“三严三实”，畅通联系服务群众“最后一公里”。深化“治庸问责”，开展电视问政、民评民议活动。加强政府党组自身建设，履行主体责任，落实“一岗双责”。严格执行领导干部经济责任审计，支持纪检监察和司法机关依纪依法查处腐败案件，形成不敢腐的震慑、扎紧不能腐的笼子、筑牢不想腐的防线。

各位代表，刚刚过去的一年，我市经济面临较大下行压力，前两季度主要经济指标增幅出现明显下滑。为此，我们稳住神、辩证看、务实干。坚持问题导向、一线工作、成效检验。加强经济运行分析，强化重点项目建设，大力开展招商引资，支持实体经济发展，统筹做好稳调促惠防等各项工作。后两季度主要经济指标回升较快，回升幅度走在全省前列，保持了经济运行“缓中趋稳、稳中向好”的态势。初步预计，地区生产总值增长8%左右；规模以上工业增加值增长8%左右；全社会固定资产投资增长18.5%；社会消费品零售总额增长11%；一般公共预算收入增长13.2%；外贸出口增长5%；实际利用外资增长10%；城镇登记失业率控制在1.5%以内；城镇居民人均可支配收入增长9%；农村居民人均可支配收入增长10%；启动62个贫困村整村推进工作，实现14万人脱贫。市四届人大五次会议确定的主要预期目标基本完成，既实现了“十二五”规划的圆满收官，又为“十三五”发展奠定了坚实的基础。

各位代表，五年的解放思想，凝聚了社会各界团结奋进、变革咸宁的强大气场；五年的务实奋斗，绘就了鄂南大地弯道超越、振兴崛起的壮丽画卷；五年的辛勤汗水，谱写了咸宁人民干事创业、追求幸福的美好乐章。成绩的取得来之不易，是省委、省政府和市委正确领导、科学决策的结果，是市人大、市政协有力支持、有效监督的结果，是全市人民努力拼搏、踏实苦干的结果，是历届市委市政府打基础、管长远的结果。在此，我谨代表市人民政府，向全市广大干部群众、离退休老同志，各民主党派、工商联、无党派人士、各人民团体，驻咸部队、武警消防官兵、公安司法干警，以及所有参与、关心和支持咸宁发展的同志们、朋友们，表示衷心的感谢并致以崇高的敬意！

五年的成就振奋人心，发展的经验尤为珍贵。最重要、最根本的一条，就是牢牢坚持发展不动摇，用绿色崛起统领经济社会发展全局，统一干部群众的思想行动。总结五年工作，我们深深地体会到：必须坚持加快发展，强化发展的主体责任，形成以发展论英雄、以业绩比高低的“精神经济学”；必须坚持抢抓机遇，把咸宁放入国际视野、国家大局、省级战略中去谋划，形成建设鄂南强市的强大动能；必须坚持创新驱动，激发市场活力，形成持续发展的有力支撑；必须坚持优化环境，加强法治和诚信建设，使咸宁成为投资创业的沃土；必须坚持改善民生，筑牢民生底线，办好民生实事，让全体人民共享发展新成果。

在肯定成绩的同时，我们也清醒地认识到，我市还处于工业化中期的初始阶段，发展的任务还很重，发展中不平衡、不协调、不可持续的问题仍然存在。我市经济规模不大，总体实力不强，继续保持经济中高速增长压力加大；支柱产业多为传统工业，处于价值链低端，极易受到冲击，而战略性新兴产业还处在培育之中，不能有效对冲经济下行压力；服务业发展不充分，消费对经济的拉动作用有限；社会事业短板较多，公共服务和保障水平较低，扶贫脱贫任务艰巨；区域发展不协调，中心城区辐射带动力不强；面对经济发展新常态，部分公务人员知识储备不足，能力不适应，责任担当意识减弱等。这些问题我们要高度重视，并努力加以改进。

二、“十三五”时期经济社会发展的总体要求

“十三五”我市面临多重叠加的战略机遇：“一带一路”、长江经济带和长江中游城市群等重大国家战略的深入实施，为我们拓展了开放发展的新空间；沿海产业转移、长江流域区域合作和对接大武汉，为我们指明了协调发展的新路径；结构性改革力度加大，“双创”工作深入推进，新型城镇化加速发展，为我们增添了创新发展的新活力；对生态文明建设的空前重视，将使我市生态优势不断放大，为我们提供了绿色发展的新思路；精准扶贫脱贫强力推进，幕阜山片区将获得更多政策和项目，为我们创造了共享发展的新机遇。国家和省“十三五”规划的实施，稳增长、调结构、补短板、防风险的具体措施出台，也将为我们带来重大机遇。

综合判断，我市仍处于发展的黄金机遇期，经济总体向好的基本面没有变，加速发展的态势没有变，发展的后发优势没有变。新的增长点正在加快孕育并不断破茧而出，新的增长动力正在加快形成并不断积蓄力量。为此，我们必须更加主动地适应经济发展新常态，更加务实地用好机遇，更加有效地推动结构性改革，强化责任担当，发扬工匠精神，奋力完成“十三五”各项目标任务。

根据《中共咸宁市委关于制定咸宁市经济和社会发展第十三个五年规划的建议》，我们编制了《咸宁市国民经济和社会发展第十三个五年规划纲要（草案）》（以下简称《纲要草案》），提请大会审议。

“十三五”时期全市经济社会发展的指导思想是：高举中国特色社会主义伟大旗帜，全面贯彻党的十八大和十八届三中、四中、五中全会精神，以马克思列宁主义、毛泽东思想、邓小平理论、“三个代表”重要思想、科学发展观为指导，深入贯彻习近平总书记系列重要讲话精神，围绕“五位一体”总体布局、“四个全面”战略布局咸宁实施，坚持创新、协调、绿色、开放、共享发展理念和以人为本、科学发展、深化改革、依法治市、统筹大局、党的领导基本原则，遵循适应、把握、引领经济发展新常态的总体要求，践行“三维纲要”和“竞进提质、升级增效、以质为帅、量质兼取”的工作方针，融入“一带一路”，开拓“一江一山”，深入实施绿色崛起战略，统筹推进小康、创新、绿色、开放、幸福咸宁建设，打造中国中部“绿心”和国际生态城市，推动发展质效在全省进位，与全省同步全面建成小康社会，为实现第二个百年奋斗目标奠定坚实基础。

今后五年全市经济社会发展的总体目标是：到2020年，地区生产总值达到1600亿元，年均增长9%左右；全社会固定资产投资年均增长14%左右；规上工业增加值年均增长9%以上；一般公共预算收入达到130亿元，年均增长10%；常住人口城镇化率55%；城乡居民人均可支配收入达到26700元。

围绕上述总体目标，《纲要草案》从经济发展、创新驱动、人民生活、公共服务和生态文明等五个方面，提出了“十三五”时期经济社会发展的31个具体目标，形成了较为完整的指标体系。这一指标体系，力求体现科学发展、绿色崛起的本质要求。

“十三五”时期，我们将建设创新能力更强、生态环境更优、开放层次更高、区域发展更协调、人民群众更幸福的新咸宁。重点是实现“五大发展”，建设“五个咸宁”。

推进供给侧改革，培育发展新动能，拓展发展新空间，完善发展新机制，使我市成为湖北改革创新试验区，实现创新发展，建设创新咸宁。加强产业整体提升和协调发展，推进幕阜山片区整体脱贫，统筹城乡发展，推动区域合作，使我市成为“中三角”合作发展先行区，实现协调发展，建设小康咸宁。优化主体功能区布局，推进生产生活绿色化，加强生态保护，健全生态文明制度，打造生态文明咸宁样板，实现绿色发展，建设绿色咸宁。主动融入“一带一路”，全面对接大武汉，构建开放发展新格局，使我市成为“万里茶道”品牌城市，实现开放发展，建设开放咸宁。增加公共服务供给，办好人民满意教育，促进更高水平就业创业，完善社会保障制度，促进社会事业进步，使我市成为独具魅力的国际生态城市，实现共享发展，建设幸福咸宁。

三、努力做好2016年工作，为“十三五”开好局起好步

2016年是全面建成小康社会决胜阶段的开局之年，做好今年的工作意义重大。要按照中央、省委、市委经济工作会议精神，坚持稳中求进工作总基调，加强供给侧结构性改革，扩大有效投资，优化供需配置，改造提升传统比较优势，培育绿色产业新优势，增强持续发展能力，推动发展质效在全省进位，努力实现“十三五”良好开局。

分析今年的形势，经济下行的压力仍然较大，我们必须克服困难、闯过关口。树立辩证思维，在看到困难和挑战的同时，更要看到机遇和优势，化危为机、危中取机，变不利为有利，在危机中实现赶超；树立底线思维，不回避矛盾，不掩盖问题，充分考虑风险和底线，做到有备无患、遇事不慌，牢牢把握发展主动权；树立竞进意识，始终保持奋发有为的精神状态，敢于啃硬骨头、敢于涉险滩，积极适应新常态，引领新常态。

今年全市经济社会发展的主要预期目标是：地区生产总值增长9%左右；规模以上工业增加值增长9%；全社会固定资产投资增长16%；居民消费价格涨幅控制在3.5%左右；社会消费品零售总额增长12.5%；一般公共预算收入增长10%；外贸出口总额增长10%；城镇居民人均可支配收入增长9%，农村居民人均可支配收入增长10%；城镇新增就业4.5万人，城镇登记失业率控制在4.6%以内；人口自然增长率控制在6.5‰以内；完成省定节能减排任务。

今年，我们将重点做好以下工作：

（一）强投资、促消费，打牢经济发展新底盘。发挥投资对经济增长的关键作用，扩大有效投资，释放新的需求，推动供给侧和需求侧双平衡。

强化有效投资。开展“重大规划项目启动年”、“推广PPP模式突破年”、“投融资体制改革深化年”三个投资主题年活动，推进项目大会战。抓紧启动咸宁通用机场、咸嘉临港综合码头及物流项目等一批“十三五”规划的重大项目。推动咸宁核电前期工作。大力推进武深高速嘉鱼北段等一批PPP模式项目建设，激活民间资本。突出抓好华中国家绿色包装物流园、通城黄龙山风电场、金盛兰冶金、立邦涂料等130个市级亿元以上重点项目。围绕国家战略和自身短板，精准谋划一批项目。积极争取国家和省预算内资金、国家专项建设基金和省长江经济带产业发展基金等。注重要素保障，做好银企对接。发挥投资委作用，坚持两月一调度工作机制，形成扩大有效投资的强大气场。

狠抓招商引资。力争全年引进2-3个世界500强企业投资项目，10个投资额10亿元以上项目，100个投资额亿元以上工业项目，完成招商引资到位资金1000亿元。依托企业、商协会及驻外机构，建立覆盖国内产业转移重点地区、逐步扩展国外招商区域的项目信息网络。充分发挥招商小分队作用，引进一批绿色环保、高新技术、高附加值的大项目、好项目。在长三角、珠三角、环渤海和香港地区精心组织经贸洽谈活动，承接发达地区的产业拓展。建立招商项目信息平台，实现市县两级信息共享。

促进消费升级。积极培育新型消费，扩大传统消费，促进消费升级。大力发展服务消费、信息消费、绿色消费、网络消费和农村消费，形成新的消费增长点。积极拓展文化娱乐、体育健身、休闲旅游等消费领域。落实国家房地产“新政”，推进购租并举，促进房地产市场健康发展。大力发展汽车消费，培育汽车后市场。统筹城乡市场建设，推进农村流通网络升级和城市商圈服务功能提升。推动“双进工程”，鼓励连锁企业设立社区超市、便利店、菜市场、家政服务等便民消费网点。落实带薪休假制度。优化服务环境，改善服务质量，强化行业自律、服务规范和监督管理，健全消费者权益保护机制。

投资是发展的主抓手。要努力营造诚信、法治、友善、亲和的投资环境，使人人成为咸宁的招商名片！

（二）调结构、转方式，增强产业发展新动能。通过做大总量、做优增量、调整存量、提升质量，加快推进新型工业化、服务业升级和农业现代化，不断优化产业结构。

做大做强工业。持续推进“工业崛起”，新增规上工业企业100家，工业技改投资增长20%。加快咸宁高新区发展，力争完成固定资产投资110亿元，规上工业增加值58亿元，实现税收13.5亿元，成功晋级国家级高新区。支持咸安环保产业园、嘉鱼长江缆索产业园、赤壁应急装备产业园、通城高新技术工业园、崇阳生物医药产业园、通山水晶文化工业园等专业园区加快发展。大力实施名牌带动、质量强市、传统产业提升、绿色新产业培育战略，加快形成绿色新产业体系。大力实施“两化融合”示范工程，重点培育20家示范企业，提升企业核心竞争力。

做精做特农业。围绕打造农产品加工千亿产业推动农业现代化。加强基地建设，重点抓好150万亩楠竹、60万亩高产油茶、50万亩生态茶园、100万亩蔬菜、50万亩精养鱼池。发挥龙头企业带动作用，重点支持羊楼洞茶业、黄袍山油茶、精华纺织、巨宁木业等行业龙头发展，申报国家级龙头企业。转变农业发展方式，坚持科技兴农，加强技术推广，走有机农业、绿色农业发展之路，促进一二三产业融合。支持农业企业抓好产品设计和标准制定，争创名优产品。支持赤壁发挥绿色产业展览交易基地品牌效应，把青砖茶打造成具有国际影响力的品牌。设立农产品加工业发展基金，推动农业企业做大做强。探索农村承包土地经营权和农村住房财产权抵押融资试点，大力发展职业农民、家庭农场、专业大户、农民专业合作社等新型主体，激活各类农业生产要素。

做优做实服务业。继续实施旅游跨越战略，丰富旅游产品，完善产业链条，提升服务质量，建设国家旅游改革创新先行区。推动国家、省级旅游休闲区和3A、4A、5A级旅游景区创建工作，力争三国赤壁古战场创5A景区取得成功，启动九宫山风景名胜区、潜山国家森林公园5A景区创建工作。以大项目为支撑，加快推动潜山“三台八景”、际华旅游目的地、温泉水上乐园、崇阳水镇文化影视城等项目建设。以“九养咸宁”为核心，加快旅游信息咨询服务平台建设，精心推出“香城泉都欢乐行”等系列主题旅游活动。加快物流园建设，重点推进高新区公铁物流园、咸安商贸物流园等项目。加快会展业发展。推进微店网、快印客、壹网时代等一批电商企业落户，支持天助网、三国鼎盛、家装e站等企业加快发展。

发展产业关键靠企业家。要形成重商、亲商的环境，让企业家显作为、有地位，扎根咸宁、融入咸宁、共享咸宁！

（三）抓机遇、优布局，拓展区域发展新空间。抢抓国家战略机遇，放大“江”的优势、发掘“山”的潜力，着力构筑“龙头昂起、南北齐飞”的市域发展新格局。

推进武咸同城化。融入武汉、发展咸宁，率先与武汉实现同城化。推动武咸快速通道天子山大桥、武深高速法泗互通和武赤线改造升级，提高咸宁至武汉段长江通航能力，积极争取武厦高铁早日立项。全面对接东湖高新区，比照实行东湖高新改革创新的优惠政策，重点发展智能机电、互联网+、现代制造、光电通讯和生命健康等绿色新产业，建设“光谷南”产业基地。加快与武汉汽车产业对接，建设嘉鱼畈湖汽车零部件产业园。优化产业布局，拓展城区北部空间，建设咸嘉绿色城镇带，把城乡主干道打造成产业经济带、绿色走廊。全力推动武咸通信同城、教育联考和旅游、交通一卡通。

建设“中三角”枢纽。抢抓长江中游城市群战略机遇，以咸岳九“小三角”为支点，全力建设“中三角”枢纽。大力推进武深高速嘉鱼北段、嘉鱼长江大桥和赤壁长江大桥建设。推进咸宁城区南外环高速、仙崇高速、咸宁至九宫山至江西永武高速公路和岳咸九铁路前期工作。加快国省干线及县乡村公路建设，推进106、107国道和208省道横路线改扩建。继续加快推进嘉鱼县临江山物流园区码头、赤壁陆水河节堤航电枢纽工程、赤壁节堤至洪庙航道整治工程等项目建设。开工建设潘家湾通用码头、中石油码头和华润电厂码头。支持通城与修水、赤壁与临湘在产业规划、招商引资、项目申报和环境保护等方面加强合作。

大力发展县域经济。发挥“北三县”沿江临港优势，建设沿江产业带和城镇连绵带。支持咸安打造改革创新试验区、绿色崛起先行区和对外开放示范区。支持嘉鱼实施临江崛起战略，打造新型临港经济区和国家级农业示范区。支持赤壁实施产业转型升级战略，打造绿色产业展览交易基地、应急装备制造基地、休闲度假基地和现代物流基地。发挥“南三县”生态绿色优势，建设幕阜山绿色产业带。支持通城打造回归创业先行区、通平修合作示范区和湘鄂赣区域边贸中心。支持崇阳建设特色资源绿色开发、产业融合发展和新型城镇化示范区。支持通山抢抓核电发展和绿色资源开发机遇，打造幕阜山绿色经济发展示范区、全国生态旅游示范县。加强市县、县县合作，探索“飞地经济”合作新模式。推动中小企业发展。支持县域经济争先进位。

机遇是发展最宝贵的财富。我们等不起、慢不得，必须在新一轮竞争中，抢抓机遇，竞进担当，推动发展!

（四）重改革、扩开放，激发创新发展新活力。以更加开阔的视野推动改革开放和创新发展，向改革要活力，向开放要动力，激发全民创新创业热情。

实施改革攻坚计划。进一步简政放权，推行权力清单和责任清单，完善市、县（区）、乡（镇）三级政务服务体系，建立全市统一的“四张清单”动态管理平台。推行电子营业执照，落实“三证合一”登记制度。深化价格改革，全面建立水、气阶梯价格制度。抓好通城农田水利设施产权制度和创新运行管护机制试点县改革。以公立医院改革为重点，推进赤壁、嘉鱼和通山医药医疗服务改革。完成咸安、通城第二批全省服务业综合改革试点区工作。借鉴崇阳改革经验，推进教育均衡发展和职业化教育。实施土地承包经营权确权登记颁证和农村集体土地“三权分置”改革，建好农村综合产权交易平台。持续深化公共服务、国有企业等重点领域和关键环节改革。加快推进政府职能转变和机构改革，继续做好事业单位分类的后续工作，强化经济发达镇行政管理体制改革试点。

全面融入“一带一路”。以“赤壁（青）砖茶”等为媒，持续推进与俄罗斯、蒙古国及中亚各国合作。筹建“法国之家”，推进友城合作，提升咸宁的开放度。推进与匈牙利在污水处理技术、温泉开发等方面合作。积极推进口岸和保税仓库建设，打造对外开放门户。大力开拓国际国内市场，组织企业参加广交会、亚欧博览会等境内外展会，鼓励企业开展跨境电子商务、海外设立营销点等新型贸易方式。着力培育外贸主体，加快推进外贸“三项工程”，支持企业出口创汇。

推进创新创业工程。建设多层次资本市场，加大直接融资比例，争取实现上市企业“零”的突破。加强与各类股权托管交易中心合作，打造区域性股权交易市场“咸宁板块”。积极推动集合债、集合贷、供应链金融等产品，引进创业基金、股权投资基金和商品交易中心等机构来咸宁发展。重点实施高新技术产业发展、专利倍增、科技服务能力提升、科技成果转化“四大工程”。鼓励企业建设工程技术研究中心、院士专家工作站。争取申报高新技术企业30家以上，创建科技企业孵化器省级3个、国家级1个，高新技术产业增加值占GDP比重达到8.5%。实施创新创业先锋计划，引进一批创新创业人才，打造一批“回乡创业园”、“电商孵化产业园”等专业园区，建设一批“绿色产业孵化园”、“产业小镇”、“众创空间”、“创业驿站”等孵化平台，力争新增孵化科技创业企业100家。

改革开放是发展最强大的动力。要大胆吸收一切成功经验和文明成果，鼓励试验，宽容失败，激励创新，崇尚成功！

（五）建品牌，抓示范，厚植绿色发展新优势。以品牌建设为抓手，加快绿色崛起步伐，让咸宁山更青、水更绿、天更蓝、城乡更美丽。

推进生态示范区建设。实施《咸宁市生态保护与建设规划》，修订完善林业生态建设、城市绿地系统、城乡绿化建设、水生态文明建设试点等专项规划。扎实推进“蓝天、碧水、净土、宁静、清洁、绿色” 创建工程。实行最严格的水资源管理，加大淦河、陆水和富水两岸生态保护，推进水生态文明示范区建设。全面开展大气污染防治，抓好主要污染物减排工作，积极发展清洁能源，推动重点行业能效提升和节能减排升级改造，争创国家级省级环保模范城市。支持通山国家绿色能源示范县建设。开展国土资源节约集约模范县（市）创建活动，推进工矿废弃地开发利用国家试点和增减挂钩省级试点工作。推广绿色建筑，发展绿色交通，营造绿色消费环境，倡导绿色低碳生活。

提升城市功能和品质。加快城市公共设施建设，推进市奥体中心（体育馆）、图书馆、档案馆、妇女儿童中心、科技馆建设。改善城市交通微循环，促进城市交通畅通。保障城市供水、供气和供电等能源安全，实现垃圾污水处理、网络通讯等设施全覆盖。推进“四城四区”提质增效。在城区北部空间开展“海绵城市”、地下综合管廊建设试点。加大棚户区改造和背街小巷整治力度，改善城乡居民住房条件和环境，使城乡居民住上更好的房子、享受更美好的环境。全面提升城市文化内涵。以“香”为魂，着力推进香产业、香文化建设，打造中国“香城”。以“泉”为介，继续办好国际温泉文化旅游节，打造中国“泉都”。以“史”为脉，建设永安阁、咸宁茶博馆等文化平台，打造“万里茶道”源头城市。建设绿道、绿地、小公园和体育休闲公园设施，加快城市客厅、城市家具建设，彰显城市人文关怀。点亮 “一山一河”，谋划月亮湾廊桥，对淦河两岸和潜山进行亮化、彩化和美化，在主城区勾勒出山水互动的美丽景观。

推进美丽乡村建设。积极推进省森林城镇、省绿色示范乡村建设，改善农村人居环境。完善乡村规划，创新管理机制，推进乡村建房规范化、有序化。推进城镇垃圾处理设施全覆盖，实现城镇垃圾焚烧处理率达到40%、污水集中处理率达到85%。完善农村生活垃圾收运处理体系，生活垃圾得到有效治理的村庄达到30%。扎实推进永久基本农田划定工作，加快高标准基本农田建设，确保19万公顷耕地保有量数量不减、质量提升。推进小流域综合治理、坡耕地综合整治、石漠化综合治理等水土保持工程，完成水利基本建设总投资10.8亿元，建设各类水利工程3972处。

绿色是发展之要、咸宁之魂。绿色决定生死。我们要像对待生命一样对待生态环境，让人民群众望得见山、看得见水、记得住乡愁！

（六）惠民生、促共享，开创社会事业新局面。人民群众是我们的衣食父母，我们要带着最深厚的感情去倾听群众呼声，了解群众疾苦，办好为民实事，让人民群众拥有更多的获得感、幸福感！

深入实施精准脱贫。加快推进“3+X”产业帮扶，力争每个贫困村至少有一个脱贫产业，每个有劳动力的贫困户至少有一个脱贫项目。整合危房改造、扶贫搬迁、移民避险解困等项目，改善贫困户住房条件，新建一批新农村建设易地移民示范居住区。深入开展“一户一技能”活动，做好就业创业帮扶，实施贫困户子女“9+3”免费教育，确保每个适龄孩子不因家庭困难而失学辍学。进一步调整新农合政策，加强贫困户重大疾病医疗救助。加强基础设施建设，统筹实施水、电、路、网、农村客运等升级工程，确保幕阜山旅游公路建成通车。进一步夯实精准脱贫工作责任，确保推进不拖延，实现脱贫见成效。

全面加强社会保障。健全促进就业创业的财政、金融、产业等政策，培育新的就业增长点。坚持以创业带动就业，加强对困难群众的就业援助。推动机关事业单位养老保险制度改革，推进职工和城乡居民全面、持续参保，推进社会保险“人口全覆盖”。进一步落实城镇职工、居民大病保险市级统筹政策。实行“先保后征”制度，建立被征地农民基本养老保险补偿机制。进一步提高城乡低保、农村五保救助标准，保障特殊困难群众基本生活。健全住房保障和供应体系，规范保障性住房建设、分配和管理。推进社会养老服务，引导民间资本投入健康养老事业。保障妇女儿童合法权益，关心和支持残疾人事业，发展公益慈善事业。

加快发展社会事业。逐步普及高中阶段教育。推进政府引导示范、社会力量为主的幼儿教育。推进市区教育一体化，落实以区为主的办学体制。全面改善农村薄弱学校办学条件，加快推进教育信息化发展。推进以提高教学质量为核心的素质教育，着力解决 “上好学”问题。加快职业教育发展。加强医疗卫生服务体系建设，抓好重大传染病、职业病防治。支持中医药事业发展。鼓励社会力量办医。做好人口计生工作，全面实施二孩政策，促进优生优育和儿童早期教育，提高人口素质。加强公共文化服务体系建设，开展文化惠民活动，鼓励文艺精品创作，发展文化产业，弘扬社会主义核心价值观。举办咸宁市首届运动会和马拉松等系列户外比赛。加快智慧城市建设，实施信息惠民工程，建设大数据中心，推进城乡光纤入户，建设城市免费Wi-Fi站点。继续做好青少年、老龄、民族宗教、广播电视、对台、档案、方志、气象、志愿者服务等工作。

提高社会治理水平。全面推进依法治市，启动“七五”普法，深化“平安咸宁”建设。加强全域网格化管理和电子监控覆盖工作，着力打造立体化、数字化的社会治安防控体系。保持打击网络诈骗、传销、涉赌、涉毒等违法犯罪活动高压态势。继续抓好易肇事肇祸精神病人、吸毒人员、刑满释放人员等特殊人群服务管理。深入开展矛盾纠纷排查，建立多层次、多途径矛盾化解机制，着力化解信访积案。严格落实安全生产责任制，完善“两化一网”体系，加强隐患排查，严防安全生产事故发生。加强食品药品安全监管，确保“舌尖上的安全”。做好征兵及双拥共建工作，统筹推进国防动员、民兵预备役、武警、应急动员等工作。推动军民融合发展。

今年，市政府继续为民办好“十件实事”：（1）新增城镇就业4.5万人，城镇登记失业率控制在4.6%以内；完成各类就业创业技能培训1.5万人。（2）全面推进精准扶贫脱贫，完成减贫脱贫9.2万人。（3）棚户区改造24414户，基本建成8260户，分配入住4086户。（4）整改中心城区47家燃煤企业，全面完成国家黄标车淘汰任务，空气质量优良率提升2个百分点。（5）关闭非煤矿山64家；开展联合整治非法采砂行动。（6）全市农村自来水普及率达到省定目标要求，在全市义务教育学校安装直饮水设备。（7）对泉塘小学、岔路口中学进行升级改造，支持碧桂园集团和省联发投新建中、小学，在100所偏远山区教学点配备教育信息化终端设备。（8）完成100家村卫生室“五化”建设，开展农村贫困妇女“两癌”免费检测。（9）建设城市主干道隔离护栏和一批港湾式公交站台，改造城区拥堵路段。（10）实施温泉中心花坛综合改造工程和银泉大道街面整理示范工程。

四、加强政府自身建设

新理念、新形势、新目标对政府工作提出了新要求。我们身上的责任重大。只有不断加强政府自身建设，才能提高履职能力和公信力。

建设法治政府。严格依照法定权限和程序行使权力，坚持法定职责必须为、法无授权不可为。健全重大决策合法性审查制度。完善政府规章立法机制，建立健全规范性文件发布和监督管理制度。规范行政执法行为，落实行政执法责任制，构建重大行政执法法制审核制度，完善责令改正等纠错机制。自觉接受人大及其常委会依法监督，积极执行人大决议决定，认真办理人大代表的议案和建议。主动接受政协民主监督，认真办理政协委员提案，广泛听取各民主党派、工商联和无党派人士意见。建立市政府常务会议学法制度，完善领导干部任前法律考试制度，开展公务人员法律知识培训，提升依法决策、依法行政、依法治理的能力和水平。

建设责任政府。自觉践行“三严三实”，以高度的责任感对待工作，以满腔的热情开展工作，以严实的要求落实工作。继续开展“万名干部进万企”活动，多为企业办实事。心系群众，善待宾客，树立友好、亲民的政府形象。开展专项整治活动，严格查处对群众、企业和基层组织的正当诉求不作为、慢作为的行为。发扬实干精神，对确定的目标任务，起而行之，锲而为之，直至成功。

建设廉洁政府。严格执行中央、省委和市委关于党风廉政建设的政策法规和要求，认真履行主体责任，推进廉洁政府建设。组织开展“主题党日+”等活动，加强理想信念、廉政警示、法规法纪教育，构建“不想腐”的自律机制。加强政府党组建设，健全工作机制，坚持重大事项集体研究，用制度管人管权管事，把权力关进制度的“笼子”，完善“不能腐”的制约机制。推进政务公开，建设阳光政府，加强公共资金、国有资产监管和领导干部经济责任审计工作，保持惩治腐败高压态势，强化“不敢腐”的惩戒机制，形成干部清正、政府清廉、政治清明的良好政务环境。

各位代表，雄关漫道真如铁，而今迈步从头越。新的目标振奋人心，新的使命催人奋进！让我们紧密团结在以习近平同志为总书记的党中央周围，在省委、省政府和市委的坚强领导下，为推进省级战略咸宁实施，建设中国中部绿心、国际生态城市，与全省同步全面建成小康社会而努力奋斗！
